# Supplementary material for: Neurotrauma clinicians’ perspectives on the contextual challenges associated with traumatic brain injury follow up in low-income and middle-income countries: A reflexive thematic analysis
Source: PLoS One. 2022 Sep 19;17(9):e0274922. doi: 10.1371/journal.pone.0274922 (PMC9484678; doi:10.1371/journal.pone.0274922)
Supplement: S1 File — (DOCX) [file pone.0274922.s001.docx]

Inclusivity in global research

PLOS’ policy on inclusivity in global research aims to improve transparency in the reporting of research performed outside of researchers’ own country or community and ensures that PLOS publications reporting global research adhere to high standards for research ethics and authorship. Authors of relevant research articles may be asked to complete the questionnaire below, which outlines ethical, cultural, and scientific considerations specific to inclusivity in global research. This questionnaire may be requested when researchers have travelled to a different country to conduct research, if research uses samples collected in another country, research with Indigenous populations or their lands, or if research is on cultural artefacts. Researchers travelling to another country solely to use laboratory equipment will not normally be required to complete the questionnaire. However, the questionnaire can be requested at the journal’s discretion for any submission – if you have been requested to complete this questionnaire by the PLOS journal you submitted to, please do so.

Please complete the questionnaire below and include this as a Supporting Information file with your manuscript. Note that if your paper is accepted for publication, this checklist will be published with your article in the supporting information files. Please ensure that you reference the checklist in the main body of your manuscript. We suggest adding a subsection ‘Inclusivity in global research’ to your Methods section and adding the following sentence: “Additional information regarding the ethical, cultural, and scientific considerations specific to inclusivity in global research is included in the Supporting Information (SX Checklist)”

The questions have been designed to be applicable to a wide range of study types, and there are subsections for both human subjects research and non-human subjects research. If any of the questions are not relevant to your research please mark them as “N/A” as appropriate.

**Ethical considerations, permits and authorship**

*This section is applicable to all research types.*

Provide details as to who granted permissions and/or consent for the study to take place in the Methods section of your manuscript. This should include the names of **all** ethics boards, governmental organizations, community leaders or other bodies that provided approval for the study. If individuals provided approval refer to these people by their role or title but do not list their name(s).

The University of Cambridge Psychology Research Ethics Committee reviewed this study (PRE.2020.010).

Reported on page number: **5**

If there were any deviations from the study protocol after approval was obtained please provide details of these changes in the Methods section of your manuscript.

Following the initial ethical approval aforementioned, two subsequent amendments were approved, including:

- Ability to recruit via social media (specifically Twitter and WhatsApp)
- Ability to conduct e-mails asynchronously via e-mail

Reported on page number: **6 & 9**

Did this study involve local collaborators that are residents of the country where the research was conducted or members of the community studied? If you do not have any authors from said communities, please provide an explanation for this below.

We were fortunate to have a number of local collaborators assist in the conduct of this study. Though the full details are omitted in this manuscript, they can be found in full in this manuscript’s accompanying protocol article in the BMJ Open journal (<http://dx.doi.org/10.1136/bmjopen-2020-041442>).

In brief: local neurotrauma clinicians of whom reside in collaborating low- and middle-income countries (LMICs) in the National Institute for Health and Care Research (NIHR) Global Health Research Group on Neurotrauma assisted as ‘gatekeepers’, forwarding information about the study to relevant clinicians in their own settings. All participants were practicing neurotrauma clinicians in LMICs. Additionally, 8 out of 14 authors on this manuscript are LMIC-residing neurotrauma clinicians.

Everyone listed as an author should meet PLOS’ criteria for authorship and all individuals who meet these criteria should be included in the author byline, rather than the acknowledgements. Authorship criteria is based on the International Committee of Medical Journal Editors (ICMJE) Uniform Requirements for Manuscripts Submitted to Biomedical Journals - for further information please see here: <https://journals.plos.org/plosone/s/authorship>.

**Human subjects research (e.g. health research, medical research, cross-cultural psychology)**

Did you obtain written informed consent from a representative of the local community or region before the research took place? How did you establish who speaks for the community? Details of written informed consent obtained from study participants should be reported separately in the Methods section of your manuscript.

All participants provided informed written electronic consent through the Qualtrics^TM^ online survey platform (Qualtrics, Provo, UT, USA).

Reported on page number: **5**

How did members of the local community provide input on the aims of the research investigation, its methodology, and its anticipated outcome(s)?

This project is part of the National Institute for Health and Care Research (NIHR) Global Health Group on Neurotrauma (GHRGN) and contributes to its strategic aims. We recognise the value of working with key stakeholders to develop research and therefore asked for peer review of this study, and its protocol, by collaborating members of the GHRGN. Their comments informed the final study design. Further, during data analysis and development of this manuscript, precursor findings were shared with all co-authors to enable critical dialogue and and provide an opportunity to add further insights or interpretations.

When engaging with the local community, how did you ensure that the informed consent documents and other materials could be understood by local stakeholders?

We recognize that ethical issues within this study include consent, confidentiality and anonymity, and data protection. Participants were deemed eligible if they have a self-declared fluency in spoken English, and the are able to provide informed consent.

Interested participants were invited to an initial telephone call to review the participant information sheet and the requirements of the study. If the physician still wishes to continue, only then was a telephone interview scheduled.

All participants were required to provide written informed consent after a cooling-off period of at least a week after the participant information sheet has been delivered. We invited potential participants to e-mail the lead authors at any time should any questions arise.

Will the findings of the research be made available in an understandable format to stakeholders in the community where the study was conducted (e.g. via a presentation, summary report, copies of publications, etc.)? Please provide details of how this will be achieved.

On completion of the study, a study summary was produced and to participants via email. A further, long-format summary of findings was produced for co-authors and international members of the NIHR GHRGN. At the time of publication, we will share results with other relevant stakeholders such as the World Federation of Neurosurgical Societies (WFNS), deliver presentations at appropriate international conferences, and host research discussions on social media platforms such as Twitter.

**Non-human subjects research using specimens/ animals collected as part of the study, or those housed in archival collections. Examples include archaeology, paleontology, botany and zoology.**

Did the permission you obtained from a local authority to perform the study include an agreement on access to outputs and benefit sharing? This may include procedures to enable fair distribution of the benefits and resources arising from the research performed. Please include any details of Prior Informed Consent and Benefit Sharing Agreements obtained. These may be required by field-specific regulations, for example the Convention on Biological Diversity (CBD) and the associated Nagoya Protocol.

N/A

If the material used in your study was imported, please A) provide the year it was imported and B) indicate whether permits were obtained to import/export the materials used, C) provide details of any permits obtained. If this information is not available, please indicate this.

N/A

If you used archival specimens, please state how the material used in your study was acquired by the institute it is held in and provide details of any permits obtained for the original excavations/ sample collection. If this information is not available, please indicate this.

N/A

How was the potential cultural significance of the materials collected in your study to local communities considered in your research design? Were Indigenous peoples and/or local researchers and institutions involved with archaeological excavations / collection of specimens? If so, please provide a description of their involvement.

N/A

If your manuscript includes photographs of human remains please indicate whether authors obtained permission from descendants or affiliated cultural communities to do so.

N/A
