# Supplementary material for: Neurotrauma clinicians’ perspectives on the contextual challenges associated with traumatic brain injury follow up in low-income and middle-income countries: A reflexive thematic analysis
Source: PLoS One. 2022 Sep 19;17(9):e0274922. doi: 10.1371/journal.pone.0274922 (PMC9484678; doi:10.1371/journal.pone.0274922)
Supplement: Attachment — Submitted filename: PlosOne 1.docx [file pone.0274922.s003.docx]

This is an interesting point addressed by the authors due to the lack of discussion regarding the challenges faced by LMIC researchers, when it comes to data collection and follow up of TBI patients. The authors performed a qualitative analysis of the perceptions of researchers from LMIC and found 5 themes. However, there are some aspects that need to be better clarified in the methods section (particularly regarding the excess of explanation and lack of HOW each phase were done). Moreover, as this is a very “dense” paper, the writing must be improved for clarity and conciseness (i.e, in lines 99 and 120, it is missing a comma; there are several wordy sentences like “the aim of inquiry “ , “through interpretation of”, “additionally at this time”, etc; there are lots of passive voice along the text – lines 111, 112, 119, 157, 189 etc).

Find bellow specific comments:

Abstract

In the conclusion: change “results in improved” to “improves”

Conclusion is confused (rewrite sentence for clarity)

Introduction

First two sentences: be more specific, showing quantitative data.

Consider rewrite this sentence for clarity: “Accurate data also facilitates the evaluation of systems and services, enabling: identification targets of wider systems improvement in injury prevention,(15) assessment of the efficacy of management decisions and patient treatment,(10,14) facilitation of quality improvement initiatives and clinical trials, and lastly, the establishment and continuation of registries; of which themselves facilitate the development of care pathways, health policy, and injury prevention strategies” - (maybe, taking of “…of which themselves… strategies”and/or splitting in two sentences)

Consider taking of “that does exist” of the sentence in lines 81 and 82

Methods

In line 111 and 113: change “may” to “might”

Typo in line 115“renumerate”, and missing comma after “participants”:

In lines 148-153: Hard to read. Consider splitting the sentence…

In line 174: please specify the demographic data collected

In lines 181-184: Hard to read sentence

Was the semi- structured interview guide pilot tested?

I think it’s better to put the initials of the authors along with the text in the following format: “B.G.S.” instead of “BGS”

Sometimes along with the methods, the authors explain and discuss about the methodology. I suggest that the authors stick to describing the methods and not justifying it. The authors published the protocol (BMJ, 2021), therefore, you might use this reference to save words.

It was hard to understand what the authors wanted to say with this sentence: “Through the use of NVivo and reflexive journal writing, a clear audit trail was created from data to findings, which also adds to the dependability and confirmability of the study”. It would be interesting if the authors could explain how they used the NVivo and the reflexive journal writing to guarantee rigour.

Results

In line 239, take the word “Unfortunatelly” off.

Please specify all the reasons for participants withdrawing from the study (and quantify objectively)

In table 2: Years of experience – 1 to 5 or 2 to 5? I thought one year of experience was an exclusion criteria.

It is interesting to be persuasive when writing the paper, however, I think it is too much. Consider taking off some words such as “important”.
